# Avian Conservation Practices Strengthen Ecosystem Services in California Vineyards

**DOI:** 10.1371/journal.pone.0027347

**Published:** 2011-11-09

**Authors:** Julie A. Jedlicka, Russell Greenberg, Deborah K. Letourneau

**Affiliations:** 1 Department of Environmental Studies, University of California Santa Cruz, Santa Cruz, California, United States of America; 2 Migratory Bird Center, Smithsonian Conservation Biology Institute, National Zoological Park, Washington, DC, United States of America; University of California, Berkeley, United States of America

## Abstract

Insectivorous Western Bluebirds (*Sialia mexicana*) occupy vineyard nest boxes established by California winegrape growers who want to encourage avian conservation. Experimentally, the provision of available nest sites serves as an alternative to exclosure methods for isolating the potential ecosystem services provided by foraging birds. We compared the abundance and species richness of avian foragers and removal rates of sentinel prey in treatments with songbird nest boxes and controls without nest boxes. The average species richness of avian insectivores increased by over 50 percent compared to controls. Insectivorous bird density nearly quadrupled, primarily due to a tenfold increase in Western Bluebird abundance. In contrast, there was no significant difference in the abundance of omnivorous or granivorous bird species some of which opportunistically forage on grapes. In a sentinel prey experiment, 2.4 times more live beet armyworms (*Spodoptera exigua*) were removed in the nest box treatment than in the control. As an estimate of the maximum foraging services provided by insectivorous birds, we found that larval removal rates measured immediately below occupied boxes averaged 3.5 times greater than in the control. Consequently the presence of Western Bluebirds in vineyard nest boxes strengthened ecosystem services to winegrape growers, illustrating a benefit of agroecological conservation practices. Predator addition and sentinel prey experiments lack some disadvantages of predator exclusion experiments and were robust methodologies for detecting ecosystem services.

## Introduction

Ecosystem services such as pest control and pollination are functions provided by biological diversity that are critical to human societies and their agricultural production [1], [2]. Nevertheless, agriculture often generates environmental pollution, contributes to habitat loss and, hence, decreases biodiversity [3], [4]. Environmentally sustainable farming practices are designed to foster biodiversity and ecosystem services. For example, bird-friendly® coffee systems are well-known for their conservation value, particularly in providing habitat for insectivorous migrant bird species [5], [6]. Studies comparing insect herbivore abundance with and without net caging over plants (exclosures) suggest that insectivorous birds significantly reduce both herbivorous arthropod abundance and plant damage in agricultural and natural systems [7], [8]. As a result, conservation of birds in agricultural landscapes may benefit growers through the provision of pest control services. For example, outside exclosures avian predation of insect pests increased quantities of marketable fruit and raised farmer income in apple [9], [10] and coffee [11], [12] production systems.

Experimental methods for quantifying ecosystem services are fraught with complications, because *in situ* manipulations (e.g. predator exclosures) can have hidden or confounding effects [13]. An alternative methodology to quantify avian predation in agroecosystems combines the manipulation of specific predator populations via the establishment of nest boxes with a sentinel prey experiment that controls for density dependent population effects. Sentinel prey studies, which monitor removal rates of immobilized, tethered, or frozen prey in the field are common in the entomology literature for comparing relative predation pressure under different conditions e.g. [14], [15], [16]. Often sentinel prey experiments are used in concert with predator abundance data to test the effects of management practices (mulching, crop diversification, plant density) on biological control by predators and parasitoids e.g. [17], [18], [19], [20] or to measure behavioral responses of natural enemies [21], [22], [23]. We know of only one experiment, however, that uses sentinel prey to quantify the activity of vertebrate predators. Perfecto et al. compared net differences in removal rates of sentinel prey (outside versus inside exclosures) in two coffee agroecosystems and found that the farm with relatively greater structural diversity had a significantly higher removal rate of prey [24]. Using vineyards as a model system, we tested for an increase in regulating services (pest removal) in agriculture by measuring sentinel prey removal with and without avian predator augmentation through the provision of nest boxes.

In California (CA), USA, grapes are the second most economically important agricultural commodity, generating over $3.2 billion US dollars in 2009 [25]. Since 1950, the expansion of vineyards has contributed to the conversion of over 1,000,000 acres of CA oak woodlands and savannas to agricultural and urban land [26], [27]. Recently the American Bird Conservancy included CA oak savannas on their list of the 20 most threatened bird habitats in the United States [28] due to the rapid conversion of breeding habitat and loss of nesting sites [29]. However, erecting nest boxes in vineyards may provide compensatory resources for Western Bluebirds (*Sialia mexicana*) [30].

The Western Bluebird, hereafter simply bluebird, is one of the species that nest in natural oak cavities and the primary occupant of vineyard nest boxes in the North and Central Coast of CA [30], serving as the focal predator species of this study. Western Bluebirds forage by perching in low vegetation and striking arthropods on the ground, air, or vegetation [31], and potentially serve as an important natural predator to many vineyard insect pest species [32]. They produce one or two broods per year between April and July and clutches usually contain four to six eggs [31]. The average energy requirement for a nine to twelve day old bluebird nestling is approximately 65 kJ per day [33]. Consequently for broods of five nestlings, about 78 g of arthropods per day must be delivered to the nest to maintain growth and development of chicks, in addition to the 23 g of arthropods per day necessary to sustain each adult bird [34].

To determine if conserving insectivorous avian predators results in increased pest control services in vineyards, we enhanced nesting opportunities for local songbird communities by establishing nest boxes in one half of two CA vineyards. By mimicking a pest outbreak in the vineyards, we investigated the response of the predator concentration treatment and control to such a perturbation. The study was designed to address the following questions: (1) How do vineyard nest boxes affect local avian abundance and composition? (2) Is avian activity restricted to the immediate location of occupied bluebird nest boxes? And (3) does the establishment of vineyard nest boxes result in increased insect pest mortality as indicated by removal rates of sentinel prey?

## Results

### Nest box Occupancy

In 2009, three avian species were the predominant occupants of vineyard nest boxes: Western Bluebirds (76.1% of box pairs), Tree Swallows, and Violet-green Swallows (*Tachycineta bicolor* and *Tachycineta thalassina* respectively, 17.4% of box pairs combined). Ash-throated Flycatchers (*Myiarchus cinerascens*) built one nest and were the only other species occupying vineyard boxes. All four species are predominately insectivorous during the breeding season.

Eggs were laid in the earliest bluebird nests in mid-April at both sites. Over the breeding season, pooling both sites, 44 bluebird nesting attempts were made. On average, each bluebird nest contained almost five eggs (mean = 4.91, SE = 0.13). Bluebird nests fledged between mid-May and late July.

### Avian Species Richness

A total of 1122 birds representing 25 species were observed at the vineyard sites (Table 1). The most common insectivorous species observed in the vineyards were Western Bluebird and Chipping Sparrows (*Spizella passerina*). Both species are associated with woodlands and savannas. Whereas bluebirds are a cavity-nesting species, Chipping Sparrows build open cup nests in vegetation, including grapevines (Jedlicka pers. obs).

**Table 1:** Total number of bird sightings by species in nest box treatments and control areas of vineyards.

Species	Latin name	Guild	Nest box	Control
Western Bluebird	*Sialia mexicana*	I	313	39
Chipping Sparrow	*Spizella passerina*	I	132	100
Tree Swallow	*Tachycineta bicolor*	I	4	0
Bullock's Oriole	*Icterus bullockii*	I	0	2
Ash-throated Flycatcher	*Myiarchus cinerascens*	I	1	1
Northern Flicker	*Colaptes auratus*	I	1	1
Black Phoebe	*Sayornis nigricans*	I	1	0
Nutall's Woodpecker	*Picoides nuttallii*	I	1	0
Yellow-rumped Warbler	*Dendroica coronata*	I	0	1
Orange-crowned Warbler	*Vermivora celata*	I	1	0
Western Tanager	*Piranga ludoviciana*	I	1	0
European Starling	*Sturnus vulgaris*	O	3	22
Brewer's Blackbird	*Euphagus cyanocephalus*	O	5	8
American Robin	*Turdus migratorius*	O	3	4
Lark Sparrow	*Chondestes grammacus*	O	1	2
American Crow	*Corvus brachyrhynchos*	O	0	2
Steller's Jay	*Cyanocitta stelleri*	O	2	0
Brown-headed Cowbird	*Molothrus ater*	O	1	0
Dark-eyed Junco	*Junco hyemalis*	O	0	1
American Goldfinch	*Spinus tristis*	G	81	150
House Finch	*Carpodacus mexicanus*	G	67	81
Wild Turkey	*Meleagris gallopavo*	G	28	21
Lesser Goldfinch	*Carduelis psaltria*	G	10	17
Mourning Dove	*Zenaida macroura*	G	4	5
California Towhee	*Pipilo crissalis*	G	0	5

Species were categorized into guilds based on the Birds of North America reference collection, where I  =  mostly insectivore, O  =  omnivore, and G  =  Granivore.

Mean avian species richness did not differ significantly, but the species richness of insectivorous birds was over 50% greater in nest box treatments than in control areas of vineyards (Table 2). This increase in the average number of insectivores per observation was due to the higher frequency of bluebird sightings and, to a lesser extent, Chipping Sparrow and Tree Swallow (Table 1).

**Table 2:** Mean (± SE) avian species richness observed or heard over the 30-minute observations and average avian abundance per 5-minute observation interval for nest box treatments and control areas.

Parameter	Nest box	Control	*P*
Avian Species Richness	4.23±0.39	3.67±0.19	0.104
Insectivore Richness	2.01±0.07	1.21±0.25	0.002
Total Avian Abundance	3.71±0.43	2.09±0.33	0.003
Western Bluebird Abundance	1.82±0.14	0.18±0.05	<0.001
Non-bluebird Insectivore Abundance	0.84±0.11	0.47±0.15	0.119
Omnivore Abundance	0.14±0.09	0.18±0.02	0.307
Granivore Abundance	1.20±0.31	1.23±0.11	0.454

Treatment means and standard errors were calculated from both sites over early, middle, and late time periods. Estimated *P*-values are from bootstrap resampling (see methods).

### Avian abundance

Total avian abundance doubled in nest box treatments early in the season and experienced a 2.6 factor increase late in the breeding season when fledglings were seen foraging with adults throughout the vineyard. Across all time periods, nest box treatments contained significantly higher avian abundances than control areas (*P* = 0.003). The increase in avian abundance in nest box treatments was driven by a single species. Western Bluebird abundance was an order of magnitude greater in nest box treatments than in control areas without nest boxes, averaging 1.8 individuals surveyed every 5 minutes compared to 0.18 individuals in control areas (*P*<0.001, Table 2). Total insectivore abundance excluding bluebirds was not significantly different across treatments (*P* = 0.119). Likewise, the abundance of both omnivores and granivores showed no consistent pattern by treatment (Table 2).

**Figure 1:**
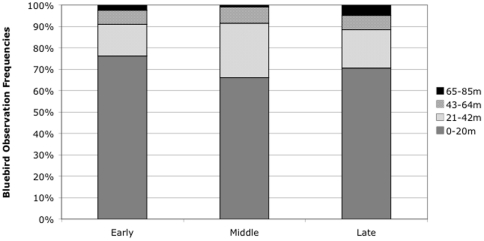
Frequency of Western Bluebird observations categorized as distance (in m) from active nest box locations during the breeding season (x-axis).

In nest box areas, the majority of the bluebirds were observed foraging near active nests (Fig. 1) and 1–5% of observations recorded bluebirds at distances over 65 m away. The number of bird detections varied over time (n = 122 early, n = 130 middle, n = 61 late season) likely because of a decreased detectability late in the breeding season due to fewer vocalizations. Bluebirds were found disproportionally closer to nest boxes early in the season, corresponding to nest building, egg laying, and incubation (Fig. 1). During the middle of the season (when first broods fledged but other nests contained eggs), bluebirds were increasingly observed at intermediate distances (21–42 m) from active nests. Late in the season proportionally more bluebirds were observed over 65 m away from active nests when bluebird adults were often seen foraging with fledglings (young that recently left the nest) in small flocks of three to five individuals.

### Sentinel Prey Experiments

**Figure 2:**
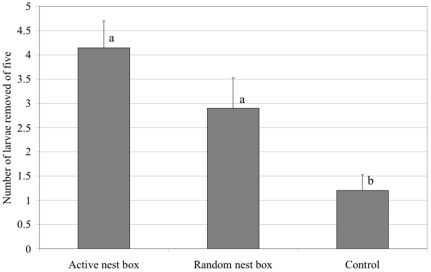
Mean number (± SE) of five lepidopteran larvae removed per transect in the pooled control (n = 10), nest box treatment (n = 10), and below active Western Bluebird nests (n = 7). Different letters indicate significant differences (*P*<0.05).

The number of sentinel larvae removed varied by treatment (control vs. nest box treatment vs. near occupied nest boxes, df = 2, X^2^ = 16.6, *P*<0.001). Those treatment effects did not vary between the vineyards (df = 1, X^2^ = 0.5, *P* = 0.48) nor was there an interaction effect between treatment and site (df = 2, X^2^ = 1.2, *P* = 0.54). Pooled removal rates of sentinel larvae were 2.4 times greater in the nest box treatment than in the control half of the vineyard (Fig. 2, n = 10 transects, mean_trmt_  =  2.9±0.6SE vs. mean_control_  = 1.2±1.0SE, z = 3.4, *P* = 0.002). The highest average removal rate of sentinel larvae occurred on transects placed within 25 m of the seven remaining active bluebird nest boxes (Fig. 2, n = 7 transects, mean = 4.14±0.6 SE larvae removed out of 5), indicating that beneficial effects of avian foraging in these vineyards can be enhanced significantly when nest boxes are occupied (larvae removed near active nests vs. control, z = 4.8 *P*<0.001). Removal rates by active nests were also higher than removal from transects placed randomly in the nest box treatments (z = 2.2, *P* = 0.066).

## Discussion

Providing songbird nest boxes in vineyards nearly quadrupled the abundance of insectivorous birds, most notably the Western Bluebird whose density increased tenfold. Nest boxes were placed in the vineyard just over one year prior to the study, however bluebirds occupied over 75% of all box pairs. Occupancy rates may further increase over time as bird populations become aware of nest box locations. Establishing nest box treatments created significant differences in avian predator densities that allowed for comparisons to baseline predator levels. Such experimental designs are advantageous because they allow for precise quantification of predator effects without the potential distortions that may be associated with exclosure methodologies [13]. One potential disadvantage of exclosures is that arthropod movement in and out of the exclosure may equalize the effects of predation pressure between experimental and control plants. This could take the form of an “osmotic effect” if protection from avian predation inside exclosures increases prey density resulting in increased prey dispersal rates away from exclosures. Structurally, the exclosure may serve to attract organisms such as web-building spiders, unnaturally increasing predation levels on other taxa within the exclosure. For-example, in a recent meta-analysis Mooney et al. found that arachnid abundance was over two times higher inside predator exclosures [8]. Each of these factors may cause an underestimation of the effects of bird predation. Furthermore, overestimates may result from a faster reproduction rate of prey inside the mesh, especially if mates are easier to find or if microclimatic conditions are favorable. Finally, exclosure studies compare presence and absence of a suite of vertebrate predators, including bats [35] and lizards [36], which makes it difficult to assess the predation effect of a particular species, or even class of predator [37].

The CA winegrape growing season overlaps with the migratory bird breeding season when, due to the energetic demands of reproductive activities, the strongest predatory pressures occur [38]. A bluebird pair with five nestlings requires 124 g of arthropods daily [34]. They produce one or two broods per year between April and July and clutches usually contain four to six eggs [31]. Data from the sentinel pest experiment during the breeding season showed a greater predation rate of larvae in the nest box treatment compared to vineyard control areas with no nest boxes. Moreover, removal rates near active nest boxes were nearly 3.5 times greater than the control. Such high predation of grapevine pests is likely a significant ecosystem service the birds provide to winegrape growers.

Bluebirds are generalist arthropod predators, preying upon insects in a range of different orders such as Lepidoptera, Orthoptera, Hemiptera, and Coleoptera [31]. As a result, bluebird presence may help provide resilience against novel pest outbreaks. The presence of new, exotic, and economically important insect pest species in United States vineyards is increasing with the notable discoveries of European grapevine moth (*Lobesia botrana,* Lepidoptera: Tortricidae) in Napa County in 2009, light brown apple moth (*Epiphyas postvittana,* Lepidoptera: Tortricidae) on the California North Coast in 2007, and glassy-winged sharpshooter (*Homalodisca vitripennis*, Hemiptera: Cicadellidae) identified in 1989 in California. Maintaining an abundant and diverse community of generalist insectivores may provide local protection against current and future pest challenges [39].

As generalist predators, bluebirds consume spiders and other arthropod enemies of herbivorous pests [31], acting as intraguild predators with uncertain net top-down trophic effects on pest levels and plant biomass [39]. Although birds may play conflicting roles as primary and secondary predators, a recent meta-analysis of exclosure studies by Mooney et al. suggests that despite their reduction of intermediate predator densities, insectivorous birds still significantly lower arthropod herbivores resulting in increased plant biomass [8].

To minimize disturbance, sentinel prey were not observed during their six hours of exposure, consequently it was not possible to determine if bluebirds were responsible for removing all sentinel larvae. Mechanical removal did not occur because humans and machinery were prevented from entering vineyard sites during the experiment. Sentinel prey were only accessible in the morning and unavailable for nocturnal predators such as bats (Chiroptera), raccoons (Carnivora: Procyonidae: *Procyon lotor*), and mice (Rodentia: Muridae). Many diurnal predators large enough to remove fastened larvae (e.g. squirrels (Rodentia: Sciuridae)) did not frequent these groomed habitats that are subject to frequent tilling and spray applications. Some larvae may have been removed by other animals such as lizards (Squamata), frogs (Anura), or ants (Formicidae). No ant swarms or evidence of larva dissection were present upon collection of transects, and no lizards were seen at vineyard sites during the entire field season. Besides housing avian predators the presence of nest boxes is not likely to influence other explanatory factors causing larvae disappearance. Both controls and predator enhancement treatments were adjacent and equidistant from wooded riparian vegetation where higher predator abundance and diversity may exist. Nevertheless the removal rate of sentinel prey in the nest box treatment averaged nearly 2.5 times higher than control areas and targeted transects below active bluebird nests resulted in 3.5 times greater larval predation than no nest box areas.

The potential for enhancing the density of insectivorous birds locally through the establishment of nest boxes, possibly increasing their population size and pest control services, is not restricted to California vineyards. As urban and agricultural expansion takes place, the popularity of bluebird trails and citizen science programs such as NestWatch (an NSF funded program run by Cornell Lab of Ornithology) has grown and bluebirds across the United States have colonized artificial nesting sites [40]. The combined range of three different species of bluebirds extends throughout the continental USA: Western, Mountain (*Sialia currucoides*), and Eastern Bluebirds (*Sialia sialis*). Therefore, USA growers will likely be able to attract breeding bluebirds wherever there are suitable habitats, including annual row crops [41]. In southwestern Germany, the cavity-nesting and insectivorous Eurasian hoopoe (*Upupa epops*) experienced strong local population declines. Stange and Havelka [42] installed nest boxes throughout vineyards and, after nine years, one hoopoe population increased from three to twelve breeding pairs. The authors concluded that providing additional nest sites and reducing pesticide applications in the area contributed to the increased population size.

Wildlife-friendly viticulture practices may be necessary to maintain breeding populations of birds in vineyards. This study was performed in organic vineyards of the California North Coast where experimental nest boxes were rapidly inhabited by breeding birds. Remnant gallery forests along the Russian River may help maintain steady food resources for nest box occupants. Other vineyard landscapes and cultural practices may not be able to recruit such high bird abundances. Further research investigating how birds use novel vineyard habitat is urgently needed as vineyards increasingly compose greater proportions of the CA landscape, often at the expense of oak savannas and woodlands. Nest boxes were placed throughout vineyard rows on existing trellises, supporting high densities of insectivorous birds. This nest box placement is suitable for many winegrape growers in the region whose machinery is built to accommodate trellises and/or who employ workers to harvest crops. Nest box placement in vineyard rows may not be feasible for highly mechanized vineyard systems where suitable box placement may be limited to the vineyard perimeter.

### Conservation Implications

In 2008, over 318,000 hectares in CA were devoted to grape cultivation [43]. Tremendous potential exists to expand avian conservation practices by increasing the numbers of songbird nest boxes in vineyards. Growers may benefit not only from the pest control services provided by breeding birds, but may also target their bird-friendly® wine to the growing organic and eco-friendly consumer markets [44]. Developing and marketing bird-friendly® wine could differentiate producers in the marketplace and empower environmentally-conscious consumers to support more sustainable production systems.

In this study, we did not monitor the conservation impact of nest box placement, but rather documented how conservation practices benefit growers. Fiehler et al. demonstrated that California vineyard nest boxes provide compensatory breeding resources for bluebirds [30]. Bluebird clutch size was larger and nest initiation date earlier in vineyards compared to neighboring oak-savanna habitat. These findings offer promise, but studies that measure the population dynamics of birds across landscapes will be required to assess the conservation potential of vineyard nest boxes throughout the state. In particular, the reproductive success of vineyard box occupants must be greater than local replacement rates. If vineyards do not serve as ‘sink’ habitats and breeding populations are sustained year after year, then the practice of providing vineyard nest boxes may be a vital component of bird conservation efforts.

Research that broadens conservation biological control to include avian predators may appear to be a novel step for Integrated Pest Management. However, these investigations resurrect a former research focus within the US Department of Agriculture (USDA) before the advent of DDT and other cheaply produced materials for pest control. From 1885 to 1940 a division of the Bureau of Biological Survey (part of the USDA) called economic ornithology was devoted to researching avian biological control [45], [46]. Our study revitalizes economic ornithology in the context of ecosystem services, and shows that the conservation practice of providing nest boxes increases the abundance of mobile, recruiting, insectivorous predators that can rapidly consume sentinel pests in contemporary, high-value crop production systems.

## Materials and Methods

### Ethics Statement

Vertebrate animals were approved for use in the study by the United States Geological Survey (Permit Number: 22665) and the University of California's Institutional Animal Care and Use Committee (Permit Number: Letod0705) and efforts were made to minimize animal suffering. Sentinel prey were approved for use by the United States Department of Agriculture's Animal and Plant Health Inspection Service (Permit Number: P526P-08-00396).

### Study sites

Vineyards chosen for this experiment were located 12 km from each other in Mendocino County, CA, USA: in Hopland (33.2 ha, 38°59′N, 123°06′W) and near Ukiah (51.4 ha, 39°04′N, 123°09′W). Both study sites were certified organic vineyards planted between 1985 and 1988. In addition to vineyards, forest remnants and wooded riparian vegetation are common landscape features in the county. Vineyard sites were both adjacent to the Russian River and managed identically by the same grower who is responsible for an additional 351.4 hectares of winegrape in the region. Chardonnay grapevines were grown on trellises forming rows. Tilling occurred in every other tractor row, alternating with cultivated cover crops - 97% clover (*Trifolium spp*), and 3% Queen Anne's Lace (*Daucus carota*). Grapevines were pruned to 6 buds per lineal foot of cordon with yields averaging 6 metric tons per acre [47]. Timing of the annual harvest is climate-dependent, but usually occurs in September and October.

### Nest box management

**Figure 3:**
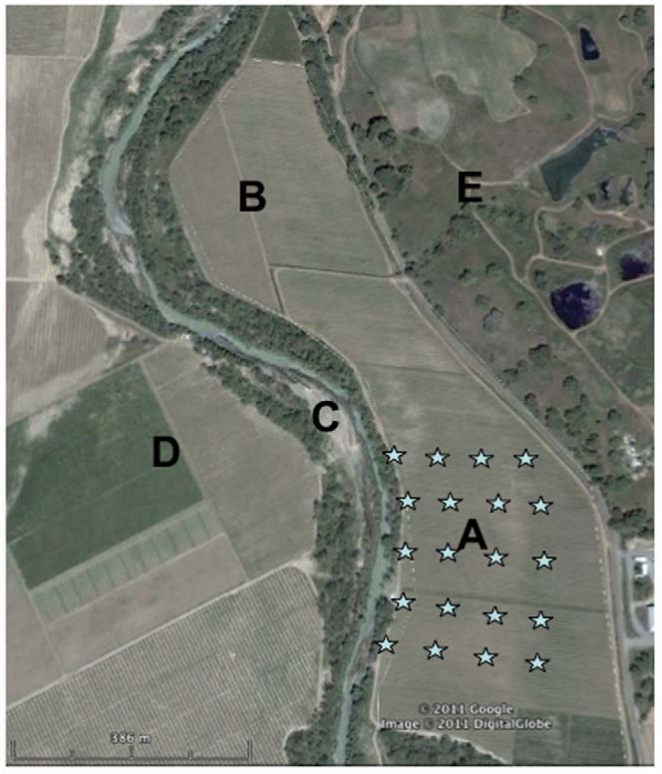
Aerial view of one vineyard site illustrating: (A) experimental treatment; (B) no nest box control; (C) wooded riparian zone; (D) surrounding vineyards; and (E) oak savannas. Within nest box treatment (A), each star indicates one pair of nest boxes mounted back-to-back 85 m from each other.

Each vineyard was divided in half, and randomly assigned either as a control or predator enhancement (nest box) treatment. A buffer of at least 250 m was left between the nest box treatment and control because nearest-neighbor distances of bluebird nests ranged from 120–240 m over a 5-year CA study [48], (Fig. 3). Nest boxes were constructed from redwood following recommendations of the North American Bluebird Society (13.9 cm by 10.2 cm by at least 23.8 cm tall with entrance hole opening of 3.8 cm diameter) [49]. Because swallow species occupy vineyard boxes and defend territories from conspecific pairs but not bluebirds, we erected boxes back-to-back in pairs within nest box treatments. Because no other bird species are common occupants of vineyard boxes, this design ensured unoccupied boxes throughout the vineyard would be available for bluebirds. In Jan 2008, nest box pairs were placed in predator treatments, spaced 85 m from each other based on nearest-neighbor distances measured by Dickinson & Leonard where a 68% nest box occupancy rate was achieved [48]. Twenty-three to 24 nest box pairs were established in a grid pattern in 5 to 6 rows (Fig. 3). Each row consisted of 3 to 6 pairs of boxes on 3.1 m t-posts placed 0.6 m into the ground along grapevine trellises. All nest boxes were cleaned of previous reproductive materials in February 2009 and checked weekly for nesting activity during the 2009 avian reproductive season from March through July. Once bluebird nests were found to contain eggs, Noel predator guards made of wire mesh hardware cloth were attached to the outside of the boxes to prevent predation by raccoons (*Procyon lotor*) or domestic cats (*Felis catus*) [50].

### Avian Observations

Avian observations were performed at five locations in both the control and nest box treatments. In both treatments, observation points were only selected if they were located at least 85 m from each other and any vineyard edges (i.e. riparian habitat and roads). In the control, observation points were selected by arbitrarily placing a finger down on maps of control areas. In nest box treatments, active bluebird nest boxes were randomly selected as observation points. Nest boxes were monitored weekly to assess bluebird reproductive activity. A nest was defined as active if it contained eggs and/or live nestlings. Abandoned nests with eggs were no longer considered active if eggs had not hatched in three weeks and no adults appeared to be entering the box.

All observations were conducted on days without strong winds or rain. Observations began shortly after sunrise and continued until approximately 10 am when avian activity decreased. Treatments were sampled on consecutive days, weather permitting. Sampling occurred biweekly at each vineyard (alternating sites between weeks) from mid-April to mid-July. The sampling order of observation points was altered each week to avoid temporal biases in observations.

At observation points in both treatments, standard avian point count procedures [51] were modified as follows. Point counts were performed from a camouflaged ground hunting blind (Ameristep one-person chair blind #403580, gandermountain.com) by the same observer (JJ). Once in position, the observer waited five minutes before sampling to minimize human disturbance. All birds seen or heard on vineyard vegetation (not flying overhead) within an 85 m radius from the observation point were recorded for one minute. Samples were repeated at five-minute intervals for a 30-minute duration at each point. Once birds were located, their species identity and distance from the observation point were recorded. Because vineyards were established in a mechanized grid where all tractor rows were 3.05 m wide, distances were relatively easy to estimate.

### Avian Classification and Justification

In California vineyards, nine of the ten avian species that occupy nest boxes of the dimension used in this study are insectivorous, with House Sparrow (*Passer domesticus*; omnivore) being the one exception [52]. In this study we focused on bluebirds because of their high nest box occupancy rate and greater likelihood to forage on vineyard insect pests. For example, swallows forage upon aerial insects over great distances [53], [54] and are not likely to be consuming pest insects from vineyard vegetation.

Avian species were divided into three guilds (insectivores, omnivores, or granivores) according to their predominant diets during the breeding season based on the Birds of North America reference collection. For example, although Chipping Sparrows (*Spizella passerina*) regularly consume seeds, they are categorized as insectivores because stomach-content analyses show invertebrates (primarily insects) to comprise the majority of their diet during the breeding season [55]. The omnivorous guild includes partial frugivores, some of which consume ripe grapes. Avian species that opportunistically forage on grape crops include the granivorous House Finch (*Carpodacus mexicanus*) and several omnivorous species such as European Starling (*Sturnus vulgaris*), Brewer's Blackbird (*Euphagus cyanocephalus*), and American Robin (*Turdus migratorius*). These potential pest species did not occupy nest boxes during the duration of this study, as some are ground or open cup nesters and others (e.g. starlings) could not fit through the box entrance hole.

### Sentinel Prey Experiment

The University of California Division of Agriculture and Natural Resources recognizes many lepidopteran species, including beet armyworm (*Spodoptera exigua,* Lepidoptera: Noctuidae), as California vineyard pests [56]. *S. exigua* eggs are laid on vineyard weeds or cover crops and larvae may feed on ground vegetation or climb up grapevines producing plant damage [56]. Fifth instars of larvae (∼12 mm long) were purchased from Bio-Serv and used for sentinel prey experiments at each vineyard site on consecutive days in June, 2009. *S. exigua* larvae were placed on the ground in transects containing five individuals pinned through their last abdominal segment to 10.2 cm^2^ brown cardboard squares, restricting the movement but not killing the insect. Each larva was placed 5m apart with cardboard squares staked into the ground in vineyard tractor rows containing cover crops. Larvae were pinned directly before placement in transects, and all sentinel pests were set out before 7:00 am. One transect, consisting of five presentation stations, was established at 10 different locations in each vineyard: at five randomly selected points in the nest box treatment, and at the five randomly selected vineyard control points chosen for avian observations. In addition, all active Western Bluebird nest boxes located at least 85 m from the riparian edge were used to quantify the maximum predatory response to sentinel prey (n = 4 and 3 at each vineyard site near the end of the season when these trials were conducted). The first larva of each transect was placed in the tractor row adjacent to the occupied box such that the final larva was approximately 25 m from the active nest. All remaining larvae were recollected approximately 6 hours later the same day and each presentation station was recorded as either present (dead from sun exposure) or missing, signifying consumption from predators. No vineyard workers or machines were present within the duration of the experiment.

### Data analysis

To reflect changes in phenology, avian observations were categorized into one of three 4-week long time periods during the breeding bird season corresponding to early (22-Apr - 22-May; birds finding territories, building nests, some with eggs), middle (23-May - 20-Jun; first broods are fledging, other nests with eggs), and late (21-Jun - 19 Jul; second broods fledging, less singing). From the avian observation data we calculated (1) the mean species richness (of all birds and strictly insectivorous birds) over the 30 min sample; and (2) the mean abundance of all birds, Western Bluebirds, and avian species divided into three guilds (insectivores, omnivores, granivores) per 5-minute observation interval. For the latter calculations, 5-minute observation means were averaged together to provide one representation of abundance per treatment at each site in early, middle and late time periods.

Avian observation data (either raw or transformed) did not meet ANOVA assumptions and were randomly resampled (with replacement) using bootstrap estimation. Means and standard deviations were calculated per time period (n = 3), treatment (n = 2) and site (n = 2). Consequently each treatment contained 6 replicates (3 time periods by 2 sites). In order to test each dependent variable against the null hypothesis of no difference between treatments, we pooled treatment means and randomly resampled 1000 means based on a sample size of six. The resampling was performed twice and the difference between these two samples was calculated to form a distribution of means representing the null hypothesis of no treatment effect. Actual differences in nest box and control means were compared to the null distribution of differences, enabling the estimation of an associated *P*-value.

In the sentinel prey experiment, number of larvae removed per transect ranged from zero to five and was analyzed with a generalized linear mixed model (GLMM) using a binomial distribution and logit link function. The full GLMM included treatment (active nests, random nest box, or control), site (n = 2), and treatment x site as fixed effects that were nested by spatial location in nest box or control areas of the vineyard (random effect). To test for effect, the full GLMM was compared to a null GLMM that was identical except that it excluded the fixed effect of interest. Full and null GLMMs were compared with an ANOVA. The GLMMs, ANOVA, and post-hoc contrasts were performed with R version 2.13 [57] and the lme4 package [58]. All other statistical tests were performed with Systat version 12.
